# On the promise of personalized learning for educational equity

**DOI:** 10.1038/s41539-023-00174-x

**Published:** 2023-08-04

**Authors:** Hanna Dumont, Douglas D. Ready

**Affiliations:** 1 Department of Educational Sciences, University of Potsdam, Potsdam, Germany; 2 Teachers College, Columbia University, New York, USA

**Keywords:** Education, Psychology, Sociology

## Abstract

Students enter school with a vast range of individual differences, resulting from the complex interplay between genetic dispositions and unequal environmental conditions. Schools thus face the challenge of organizing instruction and providing equal opportunities for students with diverse needs. Schools have traditionally managed student heterogeneity by sorting students both within and between schools according to their academic ability. However, empirical evidence suggests that such tracking approaches increase inequalities. In more recent years, driven largely by technological advances, there have been calls to embrace students’ individual differences in the classroom and to personalize students’ learning experiences. A central justification for personalized learning is its potential to improve educational equity. In this paper, we discuss whether and under which conditions personalized learning can indeed increase equity in K-12 education by bringing together empirical and theoretical insights from different fields, including the learning sciences, philosophy, psychology, and sociology. We distinguish between different conceptions of equity and argue that personalized learning is unlikely to result in “equality of outcomes” and, by definition, does not provide “equality of inputs”. However, if implemented in a high-quality way, personalized learning is in line with “adequacy” notions of equity, which aim to equip all students with the basic competencies to participate in society as active members and to live meaningful lives.

## From tracking to personalized learning

Resulting from the complex interplay between genetic dispositions and unequal environmental conditions, students begin formal schooling with diverse sets of cognitive skills and socio-emotional characteristics^1,2^ which are associated with how well and how fast students will learn. Schools around the world thus face the challenge of organizing instruction for large numbers of students while at the same time responding to their individual needs. Given that academic skills vary by student socio-economic background, how schools manage student heterogeneity influences the provision of equal opportunities and the capacity to promote educational equity.

Schools have traditionally treated academic differences as obstacles to be surmounted during classroom instruction, and sought to reduce student heterogeneity by grouping students with similar abilities together. The most obvious example of this is the worldwide practice of organizing students by age, driven by the assumption that students with similar ages have similar abilities and thus require similar instruction^3,4^. Schools also sort students according to their abilities into different courses, educational programs and schools, with the aim of creating even more homogeneous groups so that teachers can direct instruction towards the average ability level in a classroom^5,6^. However, robust empirical evidence now suggests that such tracking practices increase educational inequalities as less-demanding tracks and courses often provide less opportunities to learn, with disadvantaged students more often assigned to these lower-level tracks^6,7^.

In recent years, there have been calls to embrace students’ individual differences^8^ through personalized learning—an umbrella term for the idea of adapting instruction to students’ individual needs and providing unique learning experiences for each student. Dockterman^3^ even calls for “a new dominant pedagogy” in which “one would never expect all teachers to teach the same lesson on the same day in the same way to all students” (p.15). Technological advances together with major investments by governments, venture philanthropies and technology companies have vastly increased the availability and use of technological solutions for personalized learning in schools and thus contributed to this paradigm shift.

The increasing calls for personalized learning often go hand in hand with the premise that personalized learning will lead to more equitable academic outcomes among students of different social backgrounds^8,9^. The fact that children from low socioeconomic backgrounds are less likely to succeed in school than their peers from higher socioeconomic backgrounds continues to be a cause of concern for education systems worldwide^10,11^. Alarmingly, despite global educational expansions, socioeconomic disparities in academic achievement have remained stable over multiple generations^12^ and have even increased in many countries over the past 50 years^13^. Given these inequalities, the aim of this paper is to discuss whether and under which conditions personalized learning can increase equity in K-12 education by bringing together empirical and theoretical insights from different fields, including the learning sciences, philosophy, psychology and sociology.

## Educational equity: theoretical and empirical perspectives

As much as there is agreement on educational equity as a valuable goal, there is no agreement regarding its definition. In fact, very different and even contrasting (implicit or explicit) conceptions of equity exist among researchers, educators, and policy-makers and have been the subject of philosophical debates for decades^14–17^. In the present paper, we distinguish between prominent conceptions of equity and employ them as an analytic lens as we address the implications of personalized learning for educational equity.

One widely held notion of educational equity centers around educational outcomes, asserting that equity entails students of different backgrounds achieving equal outcomes such as academic performance^15,16^. This conception of equity, often referred to as “equality of outcomes”, is based on the premise that educational outcomes serve as a gateway to later life goods such as income, social status and health^16^. This perspective is also reflected in the way social inequalities in education are typically operationalized, particularly through SES achievement gaps, which highlight disparities in academic outcomes between students from different backgrounds.

A widespread counter-perspective to the “equality of outcomes” conception of equity argues that ensuring equality of opportunities is a more appropriate goal^15^. Complicating matters, equality of opportunity is in itself a concept with many different meanings^16^. One common understanding of equality of opportunity is the provision with equal inputs, or resources^16,17^. Schouten^16^, however, cautions that this “equality of inputs” conception “will do little more than reinforce unequal opportunities that already exist” (p. 2). In line with this concern, Sokolowski and Ansari^8^ contend that “different children require different inputs” (p. 6) to ensure educational equity.

Another conception of educational equity, known as “sufficientarianism” or “adequacy”^16–18^ takes into account both inputs and outputs. Scholars following this perspective argue that it may be morally acceptable or even necessary to treat individuals unequally by providing more resources to those who are at risk of falling short of achieving adequate outcomes. That is, the inputs provided should ensure that all students reach a minimum level of educational outcomes. Once this minimum level is achieved, any inequality in educational outcomes is seen to be no longer problematic^15^.

To gain a comprehensive understanding of how personalized learning can improve educational equity, we must also examine the role of schools in exacerbating or mitigating inequalities—a topic that has been the subject of scholarly debate for decades. A large group of scholars views schools as “sorting machines” that contribute to the (re-)production of social inequalities^19^. In contrast, considering the substantial inequalities in academic skills already present at kindergarten entry, others argue that schools serve as “great equalizers” because learning experiences in school are more equal than learning experiences out of school^20,21^. Importantly, there is robust empirical support for both arguments. While the two perspectives may seem contradictory, it is important to note that the two viewpoints employ different counterfactuals^22^. Scholars arguing that schools reproduce or even exacerbate inequality ask, “What would inequality look like if students attended identical schools?’’, scholars focusing on the potentially equalizing effects of schooling ask, “What would inequality look like if students did not attend school?’’ Therefore, when analyzing how personalized learning can improve educational equity, it is important to consider what the learning experience of students would be like without personalized learning, which may vary greatly depending on the respective socioeconomic context.

## Personalized learning: the revival of a multifaceted concept

The idea of personalizing learning by adapting instruction to students’ individual differences has existed for centuries^3,23^. Notably, Dewey’s^24^ progressive educational philosophy can be regarded a key historical root of personalized learning as he argued that education should be centered around students’ interests, experiences, and abilities. In the 1970’s, the notion of personalizing learning gained further prominence thanks to Vygotsky’s^25^ theoretical concept of students’ “zone of proximal development” and Cronbach and Snow’s^26^ empirical research on so-called “aptitude-treatment interactions”, which aimed to identify the most effective instructional strategies based on students' individual characteristics. Despite this long history in academic circles, personalization has not been widely implemented in practice, most likely because adapting instruction to individual students is highly challenging for teachers^27^. In recent years, however, technological advances have presented new opportunities to take students’ needs into account during classroom instruction, propelling the increased popularity of personalized learning^28^.

While personalized learning generally refers to the idea of responding to individual student needs during classroom instruction, it appears in many different forms^29^. In fact, the term personalized learning has been used to describe a wide range of different instructional approaches^30,31^. This lack of a shared definition is further complicated by the fact that other terms—such as adaptive teaching^32,33^, individualized instruction^34,35^, or differentiated instruction^36,37^—are often used interchangeably with the term personalized learning. One key difference between these other terms and personalized learning is that the latter is mostly used when classroom instruction involves learning technologies such as adaptive learning systems, intelligent tutoring systems or even educational robots^30,38^. Such learning technologies continuously collect data about students and adjust tasks, instructional materials or feedback accordingly via algorithms or artificial intelligence. To date, there are numerous technological solutions for personalized learning available, which differ greatly with respect to the data collected, the learner characteristics taken into account (e.g. prior knowledge, typical errors, motivation, self-regulation skills), the use of artificial intelligence and the role of teachers^39,40^ However, despite the great advantages of learning technologies for personalized learning, scholars have noted that personalized learning does not necessarily require the use of technologies^41^.

Personalized learning does find support in the recent literature, with some studies reporting that such approaches, especially those supported by adaptive learning technologies, are more effective in raising student achievement than traditional non-adaptive instruction^34,37,39,42,43^. Whereas the majority of this research occurred in high-income countries, technology-supported personalized learning has also been shown to be effective in low- and middle-income countries^29,44^. In contrast, the emerging evidence for the benefits of personalized learning to reduce educational inequalities is much more mixed: Some scholars have reported that technology-enabled personalized learning approaches particularly benefited low-performing students^45^. Others found that initially low-performing students^46^ and students with lower working memory capacity^47^ learned less than their more competent peers when using technology-enabled personalized learning systems. Experimentally addressing the question whether computer-assisted instruction can narrow the SES achievement gap, Chevalère et al.^48^ found that students from low- and high-SES backgrounds benefited equally from such instruction in comparison to conventional teacher-led instruction. Importantly, even though the SES achievement gap remained the same, low-SES students receiving computer-assisted instruction performed comparably well to high-SES students receiving conventional instruction. Given these mixed findings, we will now take a closer look at the conditions that must be in place for personalized learning to provide greater educational equity. More specifically, we will consider the conditions related to the human cognitive architecture, the self-regulatory and socio-emotional needs of students and the broader context of schooling.

## Considering the human cognitive architecture

Considering the human cognitive architecture is key to our understanding of whether personalized learning can improve educational equity. Over the past several decades, the learning sciences have compiled a large knowledge base on how people learn, in particular how complex knowledge is acquired beyond the mere memorization of facts. This research has shown that learning is a highly individual and active process, which happens through the interaction of individuals with their social environment^49–51^. Learners are not passive recipients of information; rather, they make sense of content by building a coherent and organized mental representation of that content and by integrating it with their prior knowledge^52^.

There are large individual differences between students in their learning potential^8^. Differences in learning potential mainly reflect differences in general cognitive abilities and in domain-specific prior knowledge, which individuals acquire via previous formal and informal learning opportunities^52^. Because prior knowledge in one domain is the foundation for acquiring new and more complex knowledge in the same domain, it is not surprising that students’ prior knowledge is the most important determinant of academic performance^53^. Importantly, differences in students’ learning potential are not stable, but dynamic and change over time^54^.

Psychological research suggests that instruction is most effective when these cognitive characteristics of students are continuously taken into account. That is, when content is too advanced given a students’ prior knowledge, there is a risk of cognitive overload whereby new knowledge is not learned or only learned on a very superficial level^55^. If the learning content is too easy and the learner is not cognitively challenged, learning can likewise be hampered. Hence, the targeted instruction associated with personalized learning should cognitively stimulate every student equally and result in learning gains of all students.

But what about educational equity? The answer greatly depends on the considered conception of equity. If equity is understood solely in terms of "equality of outcomes," personalized learning may have limited benefits. In fact, personalized learning, which helps all individuals reach their full potential, may accentuate and even exacerbate student differences in academic performance, as high-ability students are permitted to advance more quickly through curricular content and their initial advantage over their low-ability peers grows even wider^8,52^. And since academic achievement is often tied to socioeconomic status, personalized learning may then widen existing socioeconomic disparities in student outcomes.

The “equality of inputs” notion of equity also stands in contrast to personalized learning, as the explicit rationale for personalized learning is to treat students differently based on their respective needs. Hence, scholars who view equity as the provision of equal opportunities through differential treatments^8^, are likely to view personalized learning as a means of improving equity. Furthermore, the "adequacy" notion of equity, which aims to ensure that all students reach a minimum level of educational outcomes, also aligns with personalized learning, and we will explore this perspective further in the concluding section.

## Considering students’ self-regulatory and socio-emotional needs

Learning is more than a cognitive activity; rather, it encompasses multiple emotional, motivational and social processes^50,56,57^. During classroom instruction, it is therefore vital to continuously take students’ diverse needs into account in an integrative manner. In particular, students’ self-regulatory and social-emotional needs are constantly subject to change and deserve attention during instruction.

Students’ self-regulatory skills are key to understanding whether and how personalized learning can foster educational equity. There is robust empirical evidence showing that students with lower cognitive abilities and lower levels of prior knowledge are less capable of self-regulating their own learning process and require increased instructional support and guidance, a phenomenon known as the “expertise-reversal effect”^58^. Although technology-enabled personalized learning approaches adjust the difficulty level of instructional tasks, they typically do not take into account students’ self-regulatory skills such as the use of meta-cognitive strategies^59^. This means that they do not systematically develop such skills in students, yet they place enormous self-regulatory burdens on students, which can result in off-task behaviors and a lack of engagement for some students. This is supported by a recent study. In the implementation of one personalized learning system that required considerable student autonomy and assumed a high degree of student self-regulation, low-performing students learned less than their high-performing peers, despite the fact that each student had in fact received academic content “just right” for their skill level^46^. However, it is important to note that the problem of overburdening students does not solely apply to technological solutions for personalized learning. If teachers equate personalized learning with a “student-centered” approach in which students can choose their own learning content and are responsible for their learning process without much interaction with their teachers, the same issue arises. Research has consistently shown that all students need explicit instruction in self-regulation strategies^60^, pointing to the important role of teachers in helping students acquire self-regulatory skills or in explicitly designing educational technologies which gradually develop self-regulatory skills^61^.

Additional student characteristics that most personalized learning systems to date do not take into account include students’ motivation, goals, beliefs, interests, emotional states and personality^28,30^. Strong evidence suggests that such socio-emotional needs must be fulfilled via high-quality social interactions. Not only is learning a deeply social activity, learners require emotional safety and a sense of belonging in order to cognitively engage in learning^57^. Therefore, academic success requires that teachers build strong, supportive relationships with their students, which is particularly important for students from less-advantaged backgrounds^62^. This stands in contrast to some technology-based personalized learning approaches in which teachers are reduced to “facilitators.” In fact, Lee et al.^4^ found that many teachers in schools transitioning to a personalized learning approach heavily built on technologies were not able to build close relationships with their students. Similarly, Nitkin et al.^46^ reported that low-achieving students working with a personalized learning system learned the most in instructional modalities where they worked closely with teachers, and learned the least in modalities where they worked alone.

In summary, these findings underscore the crucial role of teachers in personalized learning, especially for students who are struggling academically or come from disadvantaged socioeconomic backgrounds. Without strong instructional and emotional support from teachers, personalized learning is likely to harm educational equity, regardless of the conception of equity that is applied. If students’ self-regulatory and socio-emotional needs are not taken into account, differences in educational outcomes will widen—as indicated by a recent study, which showed that students with higher working memory capacity benefited more from technology-enabled personalized learning than their peers with lower working memory^47^. Not only would the “equality of outcome” notion of equity be violated, but the “equality of inputs” perspective and the perspective of providing equal opportunities based on students' needs would also be compromised. As a result, this would also violate the “adequate” principle of equity and put low-achieving students and those from lower socioeconomic backgrounds at risk of not reaching the minimum proficiency level.

## Considering the broader context

The extent to which personalized learning can contribute to educational equity also depends on the broader context in which teaching and learning takes place. Students’ experiences with personalized learning can vary widely across (and even within) schools^46^. In many countries, between-school social and academic stratification is primarily the result of residential segregation, because most children attend schools close to their homes. In other countries, tracking is the main force driving the large differences between schools, including disparities in their socioeconomic composition. Hence, students from low- and high socioeconomic backgrounds often attend schools that differ greatly in the quality of education they provide. More specifically, schools serving high concentrations of economically disadvantaged students not only cater to larger numbers of children with academic and behavioral problems, they also have fewer resources and attract less qualified teachers than schools serving students from high socioeconomic backgrounds^63,64^. This confluence of factors makes it more difficult to provide high-quality personalized learning. This especially applies to technology-enabled personalized learning approaches, as disadvantaged schools may not have the necessary technological equipment, software, reliable and robust internet access or technical support staff, let alone teachers who know how to effectively integrate technology into teaching^65,66^.

The implementation of personalized learning in low-income countries is even more challenging than in disadvantaged schools in high-income countries. Many of the assumptions under which technology-enabled personalization operates do not transfer to low-income countries, such as a 1:1 child-computer ratio^67^. Although there has been a rapid expansion of school enrollments in the past decades in low-income countries, including those in sub-Saharan Africa, the majority of students still do not possess basic competencies in reading and mathematics^68^. Many schools in low-income countries, especially in rural areas, are confronted with a lack of physical infrastructure (e.g. electricity, enough classrooms, access to reliable internet) and educational resources (e.g. textbooks, computers), and are often challenged by underqualified and inexperienced teachers, high rates of teacher absenteeism, student-teacher-ratios as high as 100:1 and high levels of student poverty and malnutrition^67–69^. However, this does not necessarily apply to all schools as there is evidence that schools in low-income countries are also characterized by stark segregation and large differences in school quality^68^. Even though the implementation of personalized leaning under such harsh circumstances is extremely challenging^69^, there is robust evidence that among myriad interventions, pedagogical interventions in which teacher instruction is adapted to student needs are the most effective in increasing student performance^44^.

Taken together, the potential of personalized learning for improving educational equity may be hampered by the unequal conditions of the broader context in which teaching and learning takes places. No pedagogical strategy will be able to compensate for stark socioeconomic inequalities between schools and districts. At the same time, the role of personalized learning, in particular technology-enabled personalized learning, for improving educational equity may depend on the context itself. In industrialized countries, the overuse of technology may crowd out the benefits of human contact between teachers and students, which could be particularly detrimental for low-achieving students. In low income-countries with no universal access to schooling and high numbers of unqualified teachers, technology-enabled personalized learning may have a greater potential to improve equity than in industrialized countries because the technology may be all that is available for some students.

## Implications for the implementation of personalized learning

The potential of personalized learning to improve learning outcomes and educational equity can only be achieved as long as teachers continue to play a crucial role. Teachers can adapt to students’ needs in multiple ways, for instance by questioning, assessing, encouraging, modeling, managing, explaining, giving feedback, challenging, or making connections^70^. Instead of replacing teachers, learning technologies should empower teachers and facilitate learning and teaching processes^40,71^. New developments towards “hybrid intelligent learning technologies”, which have been developed in collaboration with teachers, offer great potential to rethink which teaching tasks can and should be offloaded to artificial intelligence—and which should remain the responsibility of teachers^40^. The further development of personalized learning technologies should also address whether and how students’ self-regulatory and socio-emotional needs can be taken into account. This implies addressing the role that students can play in co-constructing their learning experiences. Importantly, teachers should stay engaged with classroom learning activities at all times and monitor students’ learning whenever necessary. As long as personalized learning systems only adapt to students’ cognitive skills, teachers must respond to students’ self-regulatory skills and socio-emotional needs, which is particularly important for low-achieving and disadvantaged students^50^. Taken together, the effective incorporation of personalized learning technologies poses significant challenges for teachers, and this is likely to persist as these technologies continue to rapidly evolve through improved artificial intelligence.

Implementing personalized learning also requires a context-sensitive approach that takes into account the conditions under which learning occurs, particularly in low-income countries. In addition to obvious adjustments due to a lack of resources, an understanding of the local social environment is also needed. For instance, if high levels of teacher absenteeism are a problem, it is important that teachers do not feel obsolete when learning technologies are implemented, thus leading to even higher levels of disengagement. Hence, even though personalized learning holds great promise for improving school learning in low-income countries, the nature and form of personalized learning may be different than in high-income countries^67^. That is, personalized learning itself needs to be personalized.

Finally, whole-school approaches to implementing personalized learning may be particularly promising, because they also consider school organizational and institutional factors, which could otherwise hamper the potential of personalized learning. Two well-evaluated whole-school reform programs in the U.S. that were specifically designed for disadvantaged students—Success for All and the University of Chicago Charter School—have been shown to improve learning for disadvantaged students^72,73^. Interestingly, even though these programs do not call their instructional approach personalized learning, one key element is that teachers adapt their instruction to students’ needs.

## Aiming for adequacy instead of equality of inputs or outcomes

Given the theoretical considerations and presented empirical evidence in this paper, which reflect insights from multiple academic disciplines, what can we conclude about the potential of personalized learning to improve educational equity? Learning is a highly individual process shaped by each student’s unique characteristics. Adapting instruction to students’ specific needs and personalizing students’ learning experience should—at least in theory and when implemented as outlined in the previous section—lead to learning gains for all students and support each individual student in reaching their full potential. However, as explained above, this is likely not to lead to “equality of outcomes” and may even increase inequalities, because high-ability students may learn at a faster rate than low-ability students. The solution is certainly not to deprive high-ability students of appropriate learning opportunities.

Moreover, by definition, personalized learning cannot increase equity defined in terms of “equality of inputs”, as the concept of personalized learning is based on the premise that unequal inputs are needed to provide students with equal opportunities to learn. The complicated issue, then, is deciding how much inequality of inputs is reasonable and fair, particularly given limited resources. We argue that the solution may be found in the “adequacy” conception of educational equity^16–18^. That is, inequality in outcomes are tolerable if all students develop the basic competencies necessary to fully participate in society as active members and to live meaningful and fulfilling lives. This also implies that inequality of inputs is appropriate to the extent that at-risk students are provided the resources necessary to develop these competencies. In fact, in the U.S., the notion of educational adequacy is increasingly framed as a constitutional right, where all citizens deserve schooling that at a minimum provides the knowledge and skills needed for success in the modern world, and the ability to assume adult roles as active and engaged citizens^74^.

Based on the adequacy understanding, personalized learning surely serves the interests of educational equity. At the same time, this opens up a number of new questions and issues, in particular how basic competencies are conceptualized and defined. While there may be a wide agreement that all students should be literate and numerate, given their importance in shaping outcomes across the life span^75^, there may not be shared definitions of “basic”, let alone consensus on the host of more complex skills that the economy will demand in the future. Hence, the implementation of personalized learning must be accompanied by deep normative discussions on the ultimate aims of education, and its role in the production of a more equitable and just society.
